# Heteromorphic ZZ/ZW sex chromosomes sharing gene content with mammalian XX/XY are conserved in Madagascan chameleons of the genus *Furcifer*

**DOI:** 10.1038/s41598-024-55431-9

**Published:** 2024-02-28

**Authors:** Michail Rovatsos, Sofia Mazzoleni, Barbora Augstenová, Marie Altmanová, Petr Velenský, Frank Glaw, Antonio Sanchez, Lukáš Kratochvíl

**Affiliations:** 1 Department of Ecology, Faculty of Science, Charles University, Prague, Czech Republic; 2 Laboratory of Fish Genetics, Institute of Animal Physiology and Genetics, Czech Academy of Sciences, Liběchov, Czech Republic; 3 Prague Zoological Garden, Prague, Czech Republic; 4 Zoologische Staatssammlung München (ZSM-SNSB), Munich, Germany; 5 Department of Experimental Biology, University of Jaén, Jaén, Spain

**Keywords:** Homology, Karyotypes, Microdissection, Sex chromosomes, qPCR, Chameleons, Evolutionary genetics, Herpetology

## Abstract

Chameleons are well-known lizards with unique morphology and physiology, but their sex determination has remained poorly studied. Madagascan chameleons of the genus *Furcifer* have cytogenetically distinct Z and W sex chromosomes and occasionally Z_1_Z_1_Z_2_Z_2_/Z_1_Z_2_W multiple neo-sex chromosomes. To identify the gene content of their sex chromosomes, we microdissected and sequenced the sex chromosomes of *F. oustaleti* (ZZ/ZW) and *F. pardalis* (Z_1_Z_1_Z_2_Z_2_/Z_1_Z_2_W). In addition, we sequenced the genomes of a male and a female of *F. lateralis* (ZZ/ZW) and *F. pardalis* and performed a comparative coverage analysis between the sexes. Despite the notable heteromorphy and distinctiveness in heterochromatin content, the Z and W sex chromosomes share approximately 90% of their gene content. This finding demonstrates poor correlation of the degree of differentiation of sex chromosomes at the cytogenetic and gene level. The test of homology based on the comparison of gene copy number variation revealed that female heterogamety with differentiated sex chromosomes remained stable in the genus *Furcifer* for at least 20 million years. These chameleons co-opted for the role of sex chromosomes the same genomic region as viviparous mammals, lacertids and geckos of the genus *Paroedura*, which makes these groups excellent model for studies of convergent and divergent evolution of sex chromosomes.

## Introduction

Why living organisms are so variable in sex determination systems is still a matter of debate. In vertebrates, hermaphrodites and species with environmental sex determination (ESD) do not have sex chromosomes, sometimes for a long evolutionary time^1–3^, while many lineages have genotypic sex determination (GSD) with consistent sex-specific differences in genomes^1^. Sex chromosomes—the chromosomes with sex-determining loci in gonochoristic species—evolved multiple times, mostly, but not exclusively from autosomes. The first step in the evolution of sex chromosomes is the emergence of a sex-determining gene linked to one of them. Subsequent evolutionary pathways of sex chromosomes can reflect multiple adaptive and non-adaptive processes^4,5^ resulting among others in cessation of recombination, loss of functional genes and accumulation of repeats on unpaired sex chromosomes (Y and W) and their heterochromatinization. Nevertheless, although once understood as unidirectional from poorly to highly differentiated stages, it became clear that the pathways can be much more complex^5–7^, which is one of the reasons why uncovering the evolutionary history of sex determination can be challenging.

The evolutionary history of sex determination is far from being well reconstructed and still remains controversial also in such a popular group as amniotes, i.e. sauropsids (reptiles including birds) and mammals. For example, both ESD and GSD were suggested as ancestral for amniotes^8^. The reconstruction of the evolution of sex determination in amniotes is largely complicated by missing data in many lineages, past erroneous data highly influencing phylogenetic reconstructions, and problems to distinguish between the homology of sex chromosomes and independent co-options of the same genomic regions for the function of sex chromosomes^8^. Certain genomic regions have non-randomly higher tendency to turn into sex chromosomes^9^, with syntenic blocks homologous to chicken (GGA) chromosomes 17, Z and 4p (small arm of the chicken chromosome 4, homologous to the ancestral sex chromosomes of viviparous mammals, shared as the conserved region by marsupials and placentals) being among amniotes likely the most frequently co-opted as sex chromosomes^8^. It appears clear that the majority of amniotes have GSD and hence sex chromosomes. More than 80% of amniote species belong to just five highly diversified lineages with stable, independently evolved sex chromosomes: viviparous mammals, birds, iguanas, skinks and caenophidian snakes (reviewed in^10^). Within these groups, turnovers of sex chromosomes are very rare and have been found only in a few viviparous mammals (all known exceptions are from the single clade, muroid rodents^11^) and in the iguanas of the family Corytophanidae, i.e. basilisks and casquehead lizards, although corytophanids might be sister to all other iguanas^12^. Much higher variability can be found among the remaining 20% of species including lineages with ESD and likely several dozen times evolved sex chromosomes^10,13^. However, even within them, we can find lineages with stable sex determination for tens of millions of years^14–17^. Geckos and the acrodontan lineage of the clade Iguania, i.e. chameleons and agamid lizards, represent a rather exceptional wider amniote lineage with diversity in sex determination including both ESD and GSD^1,13,18^, although ESD in agamids has recently been questioned and deserves further studies^19^. More effort should be devoted to these variable lineages to get a more complex picture allowing more reliable reconstructions of sex determination in amniotes.

Although chameleons are the well-known, highly distinct clade of around 200 species^20^, their sex determination has remained poorly studied. Earlier reports of ESD in chameleons were found unreliable and/or disproved by more recent evidence. To our knowledge, sex chromosomes have been identified only in eight species of chameleons. Poorly differentiated XX/XY sex chromosomes were detected by Restriction site-Associated DNA sequencing in the velvet chameleon, *Chamaeleo calyptratus*^21^, and the sex linkage of the revealed male-specific marker was later confirmed and physically mapped to a macrochromosome also in the congeneric common chameleon, *Chamaeleo chamaeleon*^22^. On the other hand, six species of the genus *Furcifer* share female heterogamety with easily cytogenetically identifiable, partially heterochromatic W chromosomes^23,24^. Two species (*F. lateralis* and* F. oustaleti*) possess simple ZZ/ZW sex chromosomes with both Z and W being microchromosomes^23,24^. On the other hand, the partially heterochromatic W is much larger in the other four studied species (*F. bifidus, F. willsii, F. pardalis, F. verrucosus*) and uneven number of chromosomes in karyotypes in females and even in males suggest that these species have multiple Z_1_Z_1_Z_2_Z_2_/Z_1_Z_2_W neo-sex chromosomes formed by a fusion of the ancestral W with an autosome^24^. The phylogenetic distribution of sex chromosome morphology suggests that the multiple neo-sex chromosomes evolved several times within the genus *Furcifer*^24^. This situation contrasts with the general pattern in vertebrates, where multiple sex chromosomes evolve quite rarely under female heterogamety in comparison to male heterogamety^25–27^. Since the Z chromosomes are microchromosomes in all the six studied species of *Furcifer*, it was speculated that the sex chromosomes are homologous across the lineage stemmed from their last common ancestor^24^. Nevertheless, the Z chromosomes differ in shape (being bi-armed in some, but acrocentric in other species) and chromosome size is not reliable evidence for their homology. Here, we applied a combination of cytogenetic and sequencing approaches to uncover the gene content of sex chromosomes in the genus *Furcifer* and to test their homology within this genus and across outgroups. Based on a high level of heteromorphism of sex chromosomes, heterochromatic blocks on Ws, and sequence differences between Z and W uncovered by molecular cytogenetics (comparative genome hybridization)^23,24^, we expected that Z and W would be highly dissimilar in gene content and most of the Z-linked genes should be missing on W.

## Material and methods

### Studied material

We collected blood or tissue samples from both sexes of 13 species of chameleons (Table 1). Total DNA was isolated by DNeasy Blood and Tissue Kit (Qiagen), according to the manufacturer's protocol. DNA concentration and quality were estimated by the Nanodrop One Spectrophotometer (ThermoScientific). Whole blood cell cultures were prepared to obtain mitotic chromosome suspensions from two species (*F. oustaleti* and *F. pardalis*), according to our previously published protocol^28^. The experimental procedures were approved by the Ethics Committee of the Faculty of Science, Charles University, and the Committee for Animal Welfare of the Ministry of Agriculture of the Czech Republic (permits No. MSMT-8604/2019-7 and No. 22486/2023-4) and were performed in accordance with the relevant guidelines and regulations. For experimental design, we followed the recommendations of ARRIVE guidelines (https://arriveguidelines.org).

**Table 1:** Specimens, *per* sex and species, analyzed in the current study.

Species	Male	Female
*Brookesia therezieni*	1	1
*Calumma glawi*	1	1
*Calumma parsonii*	1	1
*Chamaeleo calyptratus*	1	1
*Furcifer campani*	1	1
*Furcifer labordi*	1	1
*Furcifer lateralis*	1	1
*Furcifer oustaleti*	1	2
*Furcifer pardalis*	1	1
*Furcifer rhinoceratus*	1	1
*Furcifer viridis*	1	1
*Kinyongia boehmei*	1	1
*Trioceros johnstoni*	1	1

### Microdissection of sex chromosomes and comparative painting

We microdissected the Z_1_ chromosome of *F. pardalis* and the Z and W chromosomes of *F. oustaleti* and sequenced the chromosome material in Illumina platform in order to identify their gene content. This approach was previously successfully applied in other reptiles^29,30^. For the microdissection, we used a Zeiss Axiovert S200 inverted microscope (Oberkochen, Germany) equipped with an Eppendorf TransferMan NK2 mechanical micromanipulator (Hamburg, Germany). 15–20 chromosomes of each type were microdissected by sterile glass needles. The microdissected chromosomal material was amplified by degenerate oligonucleotide-primed PCR (DOP-PCR), following the protocol of Marchal et al.^31^. According to chromosome morphology, the Z_1_ chromosome of *F. pardalis* is homologous to the ancestral Z chromosome of the genus *Furcifer*, while the Z_2_ chromosome is a neo-sex chromosome^23^.

We proceeded with chromosome painting in order to confirm that the amplified microdissected material includes genomic regions from the desired chromosome and that we can exclude significant contamination by material from other genomic regions. Therefore, part of the amplified material was labelled with Spectrum Orange-dUTP (Abbott) by an additional DOP-PCR and hybridized to chromosome spreads following the protocol of Marchal and colleagues^31^. In situ hybridization images were captured by an Olympus BX51 fluorescence microscope equipped with an Olympus DP70 digital camera. The images were further processed by Olympus DP manager imaging software.

Part of the amplified microdissected material was sequenced by Illumina platform with 100 base pairs (bp) pair-end option (DNA-seq) by Macrogen (South Korea), in order to identify the sex chromosome gene content. The raw Illumina reads are available in the NCBI database (BioProject PRJNA1027145). Adapters and low-quality bases were trimmed using Trimmomatic v0.39^32^ with default parameters and reads shorter than 50 bp were removed from further analyses. The trimmed Illumina reads were mapped with Geneious Prime v2022 (https://www.geneious.com) to a reference dataset of 174,674 exons, extracted from the *Anolis carolinensis* genome project^33^, the closest species to chameleons with a well assembled and annotated genome. Mapping parameters are provided in Table S1.

### Gene coverage analysis in *F. lateralis* and *F. pardalis*

In addition to chromosome microdissection, we revealed the Z-specific genes in *F. lateralis* and Z_1_-specific genes in *F. pardalis* by comparative gene coverage analysis. In female heterogametic systems with degenerated W, the ZZ males have two copies of autosomal, pseudoautosomal and Z-specific single-copy genes *per* diploid cell. On the contrary, ZW females have two copies of autosomal and pseudoautosomal single-copy genes, but only one copy of Z-specific single-copy genes. This difference between sexes is reflected in the output of reads obtained from the Illumina next generation sequencing. Z-specific single-copy genes should have half read coverage in the ZW females in comparison to ZZ males, while autosomal and pseudoautosomal genes should show equal read coverage in both sexes. This method was previously applied to several species of reptiles, including skinks^10,34^, snakes^35^, softshell turtles^36^ and geckos^17,37^.

Total DNA from one male and one female of *F. lateralis* and *F. pardalis* were sequenced using the Illumina HiSeq2500 platform, with 150 bp paired-end option (DNA-seq) at Novogene (Cambridge, UK). The raw Illumina reads are available in the NCBI database (BioProject PRJNA1027145). The Illumina reads were trimmed for adapters and low quality bases, and mapped to the reference dataset of exons from *Anolis carolinensis*, using the same parameters and software as described above for the microdissected material. From the mapping reports, we calculated the average read coverage of each gene *per* sex and species, normalized to the mode coverage. Exonic sequences with extremely high or extremely low coverage were excluded from the analysis (i.e., those with threefold difference coverage from the mode), in order to filter out genes with paralogs and pseudogenes, which might be located in multiple chromosomes, and exons contaminated with repetitive elements.

### Validation of Z-specific genes and test of sex chromosome homology by qPCR

We designed primers for putative Z-specific genes revealed by the sequencing of microdissected chromosomes and the coverage analysis. These genes were tested by qPCR for Z-specificity, following a methodology previously described by Nguyen et al.^38^ and Rovatsos et al.^39^. In the same reasoning as for comparative coverage analysis, the ZW females have half copies of Z-specific genes than ZZ males per cell, and this difference in Z-specific genes can be detected by comparing the qPCR quantification values between males and females. In addition, the same qPCR method can be applied to closely related species to reveal if the tested genes are also Z-specific, and subsequently, if a group of species share homologous sex chromosomes. This approach was successfully applied to validate genes linked to sex chromosomes and test the homology and age of a sex determination system in several species of reptiles, including geckos^17,37,40^, iguanas^41,42^ and monitors^15^.

Primers for putative Z-specific genes were designed by Primer-Blast software^43^ and Primer 3^44^. All the primers were tested first by PCR to screen for successful amplification. Per each set of primers, the PCR mix included 1 µl of DNA, 1 µl of forward and 1 µl of reverse primers (10 pmol/μl), 5 µl of 10 × PCR buffer (Bioline), 2.5 µl MgCl_2_ solution (50 mM), 1 µl of nucleotide mix (2 mM each of dATP, dCTP, dGTP, dTTP) (Roche), 0.5 μl of BioTaq DNA polymerase (5 U/μl, Bioline), and water up to a final volume of 50 µl and the PCR reaction was run at the following conditions: initial denaturation at 94 °C for 3 min, then 35 cycles at 94 °C for 30 s, 56 °C for 30 s, and 72 °C for 15 s, and final extension at 72 °C for 5 min. The PCR products were evaluated by visualization in 1% agarose gel, stained with GelRed (Biotium). Successfully amplifying primer pairs were tested by qPCR. The qPCR was performed on a LightCycler II 480 (Roche Diagnostic). The reaction mix included 2 ng of genomic DNA, 0.3 µl of each forward/reverse primer (stock solution 10 pmol/μl) and 7.5 μl of SYBR Premix Ex Taq II (Takara Bio) and water up to 15 μl of the final volume. Each sample was run in triplicates. The cycler conditions were: initial denaturation at 95 °C for 3 min, 44 cycles at 95 °C for 15 s, 56 °C for 30 s and 72 °C for 30 s. After the amplification cycles, the melting curve analysis was performed. The qPCR quantification values (crossing point—cp) were calculated by the LightCycler 480 software (version 1.5.0, Roche), using the second-derivative maximum algorithm. qPCR reactions with unspecific amplicons were excluded from further analysis. Firstly, we calculated the normalized quantification R = 2 ^Cp*mecom*^/2^Cp gene^ for each gene and specimen, and then we calculated the relative female-to-male gene dose ratio (r) for each gene, following the equation: r = R_female_/R_male_^39^. The relative gene dose ratio (r) is expected to be approximately 1.0 for autosomal and pseudoautosomal genes and approximately 0.5 for Z-linked genes.

The primer pairs that showed Z-specific relative gene dose ratio in *F. lateralis* or *F. pardalis* were also tested in additional 11 species of chameleons. If the same genes are Z-specific in additional chameleons, we assume that this group share a homologous sex determination system and subsequently, we can estimate that the age of the system is at least equal to the age of their last common ancestor. Although we are aware that alternative topologies were presented in Tonini et al.^45^ and Mezzasalma et al.^46^ for some clades, we follow phylogenetic relationships according to Pyron et al.^47^ and Tolley et al.^48^. Nevertheless, the monophyly of the genus *Furcifer* is supported in all above-mentioned phylogenies. Molecular dating data are available in Tolley et al.^48^, Zheng and Wiens et al.^49^ and Pyron^50^.

## Results

### Microdissection of sex chromosomes: chromosome painting and Illumina sequencing

**Figure 1:**
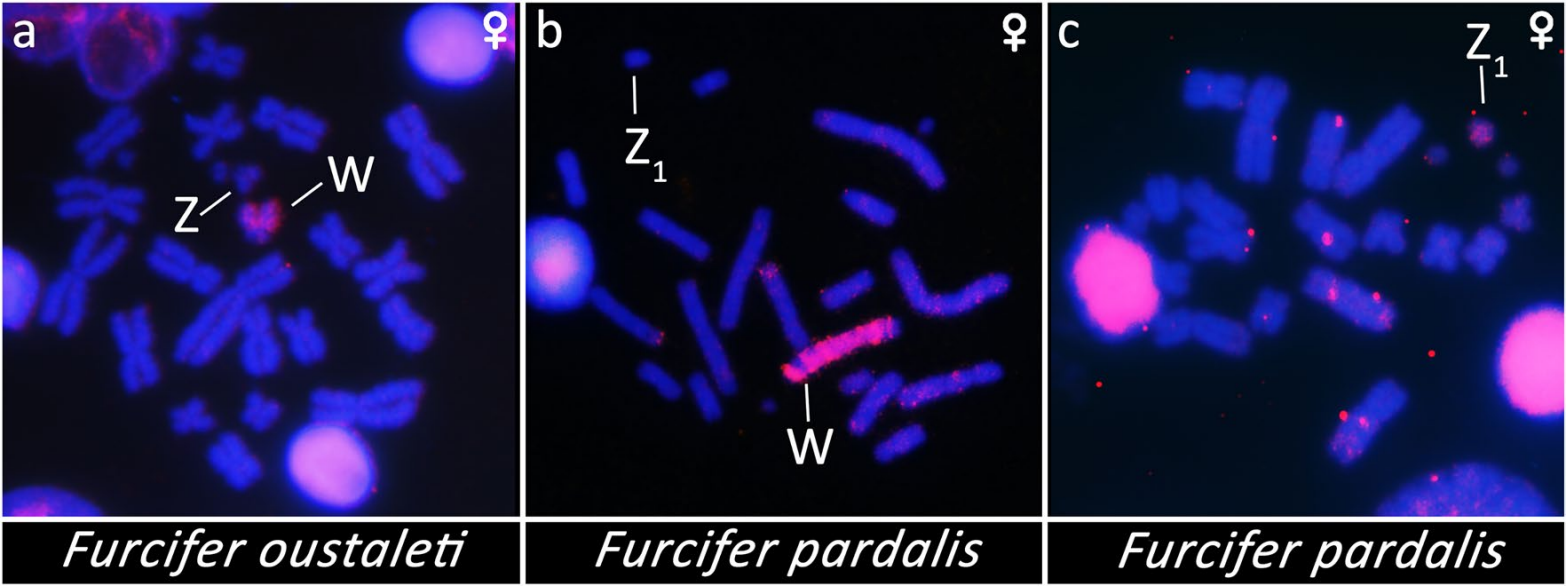
Chromosomal painting with probes from microdissected chromosomes. The probe derived from the W chromosome of *F. oustaleti* hybridized to metaphases from females of (**a**) *F. oustaleti* and (**b**) *F. pardalis*. (**c**) The hybridization of the probe from the Z_1_ chromosome of *F. pardalis* to a female metaphase of the same species. Sex chromosomes are indicated.

The probes for the Z_1_ chromosome of *F. pardalis* and W chromosomes of *F. oustaleti* strongly hybridized only to the expected chromosomes in chromosome spreads of the same species (Fig. 1), which indicates that the microdissected material has not significant contamination from other genomic regions. The probe derived from the microdissected W chromosomes of *F. oustaleti* hybridized as expected to a single chromosome (W chromosome) in chromosomal spreads of females of *F. oustaleti* (Fig. 1a) and *F. pardalis* (Fig. 1b). The W-specific probe derived from *F. oustaleti* covered a large part of the W chromosome of *F. pardalis*, which probably reflects a spread of W-specific repeats to the neo-part of its W chromosome. The probe derived from the microdissected Z_1_ chromosome of *F. pardalis* hybridized to this chromosome as expected (Fig. 1c). We did not prepare a probe for the Z chromosome of *F. oustaleti* due to the low concentration of the amplified material, which was used only for sequencing.

**Figure 2:**
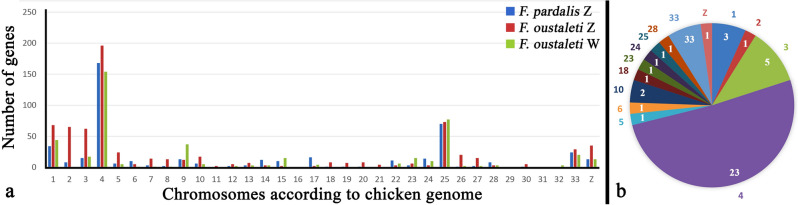
Analysis of gene content of the microdissected chromosomes. Z_1_ chromosome of *F. pardalis*, and Z and W chromosomes of *F. oustaleti* are enriched in genes with homologs linked to several chicken chromosomes, particularly 1, 4, 25, 33 and Z (**a**). The vast majority of these genes are probably pseudoautosomal, 42 genes with homologs linked mainly to chicken chromosome 4 could be Z-specific, because they were detected in both Z chromosome of *F. oustaleti* and Z_1_ chromosome of *F. pardalis*, but not in the sequence of the microdissected W chromosome (**b**). Outer numbers correspond to chicken chromosomes, inner numbers to number of genes (**b**). All data are included in Table S2.

The analysis of the gene content of the microdissected Z_1_ chromosome of *F. pardalis*, and Z and W chromosomes of *F. oustaleti* revealed that all three chromosomes are enriched in genes with homologs linked to several chicken chromosomes, particularly chromosomes GGA 1, 4, 25, 33 and Z (Fig. 2a; Table S2). The vast majority of these genes are detected in both Z and W chromosomes of *F. oustaleti*, which indicates that they are probably pseudoautosomal (Fig. 2a; Table S2). However, 42 genes could be Z-specific, because they were detected in both Z chromosome of *F. oustaleti* and Z_1_ chromosome of *F. pardalis*, but not in the sequence of the microdissected W chromosome of *F. oustaleti* (Fig. 2b). Notably, 23 out of the 42 putative Z-specific genes have homologs linked to GGA 4, located mainly in the small arm of this chromosome (GGA 4p). The analysis also supports the hypothesis that the Z_1_ chromosome of *F. pardalis* is homologous to the Z chromosome of *F. oustaleti*.

### Genome coverage analysis

**Figure 3:**
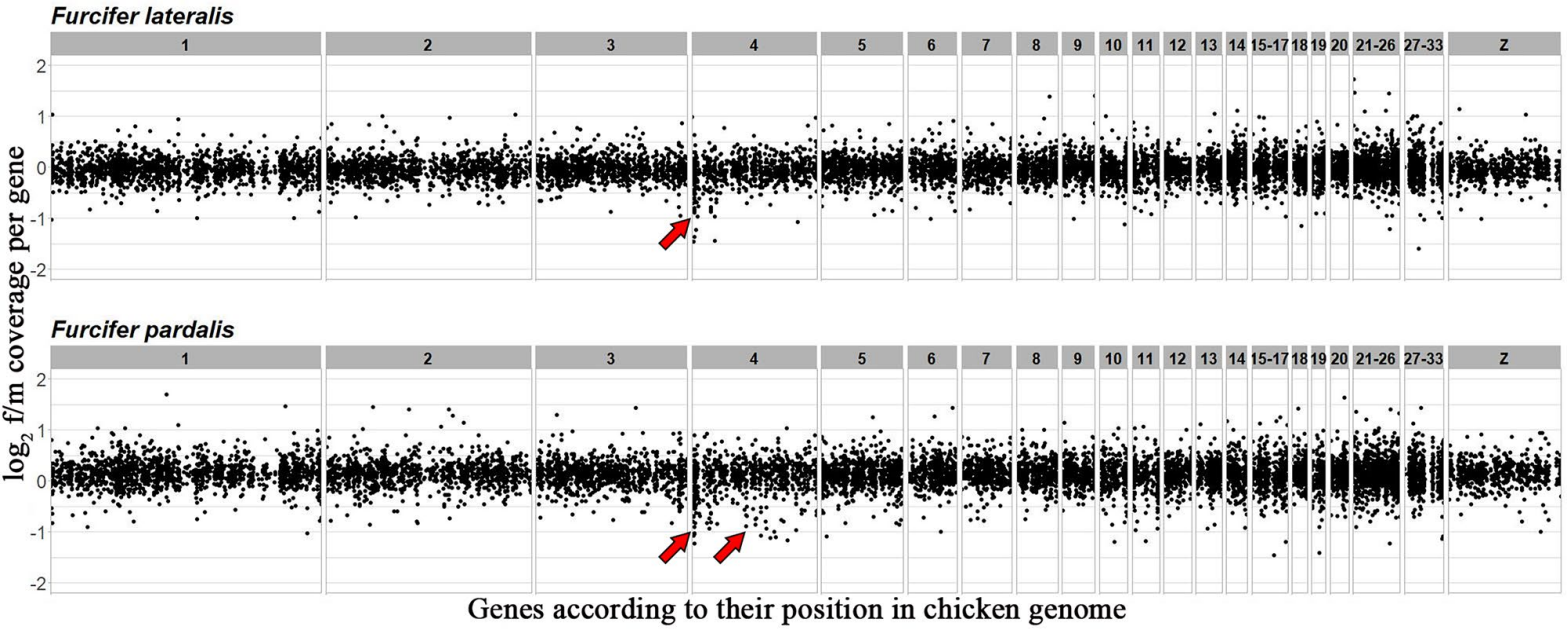
Log_2_-transformed female to male ratios of DNA-seq read coverage per gene in *F. lateralis* and *F. pardalis*. Genes are illustrated based on the position of their orthologs in the chicken genome. The Z-specific genes are expected to show half female to male read coverage ratio (log_2_-transformed ratios of ∼ − 1.00) than autosomal and pseudoautosomal genes (log_2_-transformed ratios of ∼0.00). The genomic regions with putative Z-specific genes homologous to chicken chromosome 4 are indicated by arrows. All data are included in Table S3.

The Illumina reads from the male and the female were successfully mapped to 16,947 and 16,909 genes in *F. lateralis* and *F. pardalis*, respectively. The comparative read coverage analysis revealed 371 and 353 genes with the female-to-male ratio between 0.35 and 0.65, which is close to the expected ratio for Z-specific genes. Orthologs with known chromosome positions in the chicken genome were found for 110 and 125 of them. The orthologs were linked mainly to GGA4p (Fig. 3; Table S3). In addition, this analysis further supported that the Z-specific region with a highly degenerated counterpart on W is rather small in the genus *Furcifer.* Notably, both analyses, i.e. the comparative genome coverage and the sequencing of the microdissected sex chromosomes, revealed that a genomic region homologous to GGA 4p is Z-specific in the examined species of *Furcifer*.

### Validation of putative Z-linked genes and study of homology by qPCR

**Figure 4:**
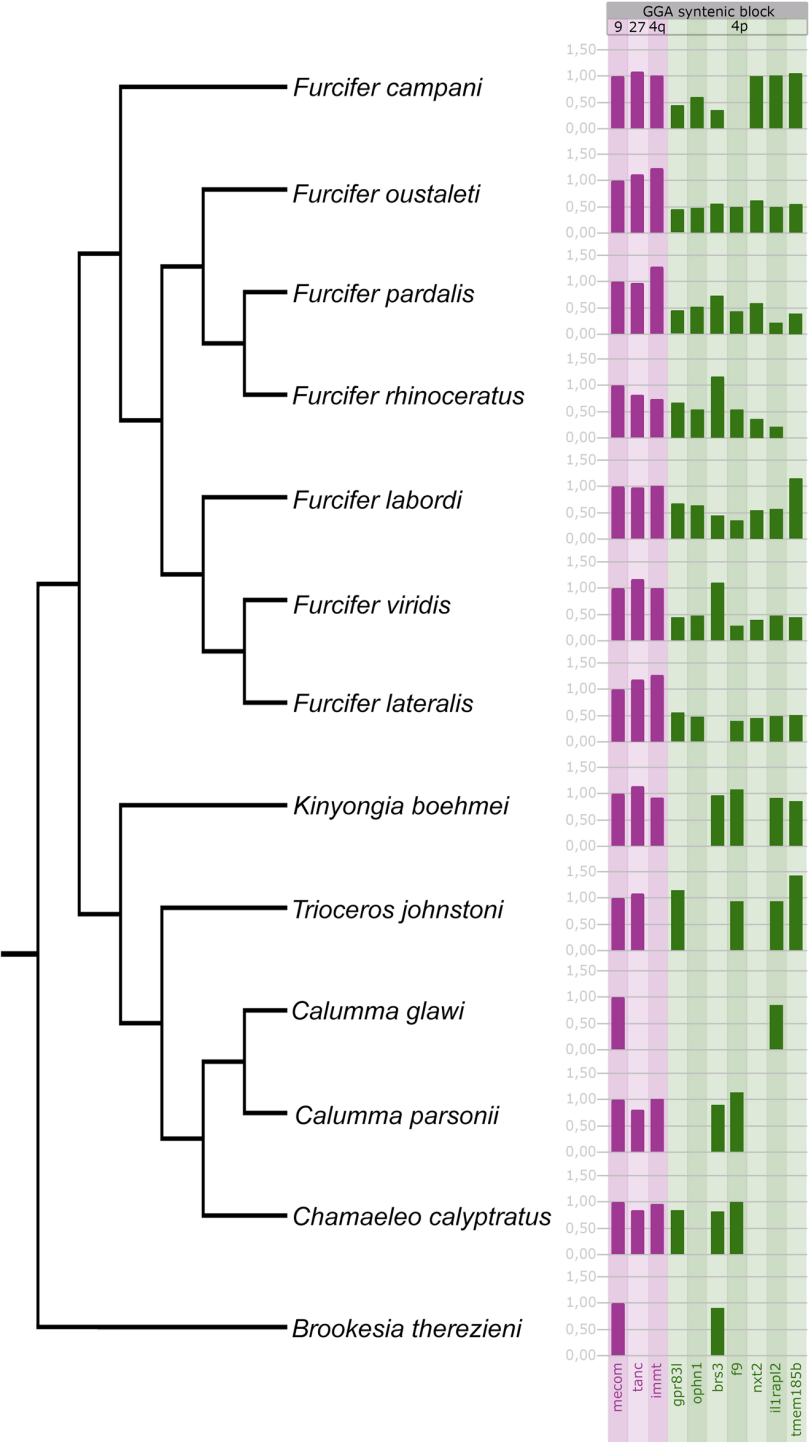
Mean relative gene dose ratios between female and male genomes in chameleons. Value 1.0 is expected for autosomal or pseudoautosomal genes, while 0.5 is consistent with Z-specificity. All data are included in Table S4. Phylogenetic relationships follow Pyron et al.^47^ and Tolley et al.^48^. Please note that alternative topologies among species are presented in Tonini et al.^45^ and Mezzasalma et al.^46^. Nevertheless, the monophyly of the genus *Furcifer* is supported in all above-mentioned phylogenies.

We used primers for three autosomal genes, namely *mecom* (GGA 27), *tan*c (GGA 9) and *immt* (GGA 4q), previously published by Rovatsos et al.^51^, for control and normalization of the qPCR values. In addition, we designed primers for seven putative Z-specific genes, namely *brs3, f9, gpr83l, il1rapl2, nxt2, ophn1*, and *tmem185a*, linked to GGA 4p, selected from the datasets of the microdissected sex chromosomes (Table S2) and the comparative coverage analysis (Table S3). The qPCR test validated the Z-specificity of these genes in *F. lateralis* and *F. pardalis* (Fig. 4; Table S4).

The same primers were tested by qPCR in additional five species of *Furcifer* (*F. campani, F. oustaleti, F. labordi, F. viridis* and *F. rhinoceratus*) (Fig. 4; Table S4). With few exceptions, the tested putative Z-specific genes showed a pattern consistent with Z-specificity in the species of the genus *Furcifer*. However, values close to 1.0 or higher were recorded for *brs3* in *F. viridis* and *F. rhinoceratus*, for *tmem185a* in *F. labordi* and *F. campani* and for *nxt2* and *il1rapl2* in *F. campani* (Fig. 4; Table S4). These exceptions could be explained by chromosomal rearrangements that occurred after the emergence of the sex determination system and during the species diversification, changing the topology of these genes to either pseudoautosomal or autosomal position.

We tested the same set of genes in six species of chameleons from the other five genera (*Calumma, Chamaeleo, Kinyongia, Trioceros, Brookesia*). Here, fewer primers were successfully amplified by qPCR, which can be expected by their larger phylogenetic distance. However, the successfully used loci did not reveal a pattern consistent with Z- or X- specificity (Fig. 4; Table S4).

## Discussion

For the first time, we uncovered the partial gene content of sex chromosomes in the chameleons of the genus *Furcifer*. The genome of a male *F. pardalis* was recently sequenced and assembled in chromosome level, resulting in 11 scaffolds, but sex chromosomes were not identified^52^. A local Blast search^53^ of the Z-specific genes revealed by coverage analysis and the Z/W-linked genes revealed from sequencing the microdissected chromosomes allowed us to assign the Z_1_ chromosome to the scaffold no 10 of the assemblies published by Xie and co-authors^52^. This assignment is further supported by previous cytogenetic analysis, where the Z_1_ chromosome was identified as the 10th pair of the male karyotype^23^.

The comparative analysis of differences in sex-specific genes across chameleons (Fig. 4; Table S4) and the previous knowledge on XX/XY sex chromosomes in the genus *Chamaeleo*^21,22^ suggest that there was a turnover of sex chromosomes or their two independent origins within chameleons in the past 45 million years (dating following^49^). At the same time, our analysis confirms that the minimal age of the sex chromosomes of the *Furcifer* chameleons is around 20 MY, i.e. the estimated age of the split between *F. campani* and the other members of the genus *Furcifer* according to the molecular dating analyses in Zheng and Wiens^49^. In spite of numerous interchromosomal rearrangements leading among others to likely repeated origins and/or disappearances of multiple neo-sex chromosomes^24^, the genus *Furcifer* can be viewed as a lineage with stable sex chromosomes. Within acrodontan iguanians, stable sex chromosomes were found also in the genus *Pogona* and their relatives (their minimal age was estimated to 25 MY^8^), demonstrating that even within a group expected to have highly variable and unstable sex determination systems, there are sublineages with rather stable sex chromosomes.

An important issue in the research of sex chromosome evolution is a quest for a proper metric of the degree of their differentiation^54^. Traditionally, sex chromosomes were categorized based on differences in morphology (size and shape) as heteromorphic (from Greek words *ἕτερος*, meaning “another”, also in the meaning “different” and μορφή, meaning: form, shape) and homomorphic (from Greek prefix ὁμός, meaning “same, identical”). At the times of classical cytogenetics, only chromosome morphology could be studied and these terms came from this era. However, up to now, the term “heteromorphic” is still used as a synonym for “highly differentiated” and “homomorphic” as poorly differentiated. Nevertheless, it is imprecise as morphology and DNA sequence differentiation do not necessarily go together. Homomorphic sex chromosomes can be largely sequentially differentiated as in mosquitos^55^ and lacertid lizards^56,57^. On the other hand, heteromorphic or otherwise cytogenetically easily distinguishable sex chromosomes can be poorly differentiated in gene content. For example, eublepharid geckos *Coleonyx elegans* and *C. mitratus* have multiple X_1_X_1_X_2_X_2_/X_1_X_2_Y neo-sex chromosomes, with the Y chromosomes being the only metacentric in the complement, yet, the sex chromosomes differ in presence of only a small number of genes^58^. Similarly, the W chromosome of the bearded dragon (*Pogona vitticeps*) is notable for heterochromatin accumulation and accumulations of repeats^18^, but its Z and W share a lot of genes and its sex determination might be controlled just by epigenetic modifications of W^59^. Therefore, we recommend using the terms hetero- and homomorphic only for the description of differences in size and shape without any connotations for the degree of differentiation at the DNA sequence levels.

The chameleons of the genus *Furcifer* share most genes between Z and W, although these chromosomes are cytogenetically easily identifiable (Figs. 1, 2). Although the sex chromosomes in the chameleons of the genus *Furcifer* are highly heteromorphic and their W chromosomes contain a notable heterochromatic block and are enriched in repeats (^23^this study), the Z and W sex chromosomes of *F. oustaleti* (Fig. 1a) share most of the gene content as revealed by the analyses of the gene content of its microdissected chromosomes (Fig. 2; Table S2). In agreement, the coverage analysis in *F. lateralis* (Fig. 3a) and *F. pardalis* (Fig. 3b) pointed to only a relatively few genes missing on their W in comparison to Z chromosomes. This finding contradicts our expectation that the heterochromatic W in chameleons of the genus *Furcifer* should lose a significant part of its gene content as in lacertids^60^ or caenophidian snakes^51^. In future, it will be interesting to explore the repeatome of the W chromosome and to compare it to accumulations of transposons and satellite DNA motifs in independently evolved W chromosomes of other lineages of reptiles, e.g. caenophidian snakes and lacertids^61,62^. The sex chromosomes in the chameleons of the genus *Furcifer* contain the same genomic region partially syntenic with the GGA4p as the ancestral XX/XY of the therian (viviparous) mammals, and ZZ/ZW sex chromosomes of the lacertid lizards and of the geckos of the genus *Paroedura* (reviewed by^8^). These clades are separated by numerous lineages with other sex determining systems (ESD and GSD with non-homologous sex chromosomes) strongly suggesting that the sex chromosomes in these four lineages evolved independently. The groups with the independent co-option of the same region can serve as a great model for evolutionary studies of convergent and divergent processes related to sex chromosome evolution under the control of the genomic background, largely gene identity and function^36^. An interesting question is why the same region homologous to GGA4p turned at least four times independently to sex chromosomes among amniotes. It was suggested that the repeated co-option of genomic regions to the role of sex chromosomes could reflect the inclusion of genes with a potential to be recruited as a sex-determining locus^9^. In GGA4p, the promising candidates might be genes homologous to *sox3* (SRY-box transcription factor 3), which next to therian mammals became a sex-determining locus in a fish^63^, or *ar* (androgen receptor), which plays a role in the sex determination of the Japanese frog *Rana rugosa* ^64^. Future research should be devoted to uncovering sex-determining genes in the genus *Furcifer*.

### Supplementary Information

Supplementary Legends.

Supplementary Table 1.

Supplementary Table 2.

Supplementary Table 3.

Supplementary Table 4.

## Data Availability

The raw Illumina reads are available in the NCBI database (BioProject PRJNA1027145). All other data are presented in the figures and tables of the manuscript.
