# Supplementary material for: Heteromorphic ZZ/ZW sex chromosomes sharing gene content with mammalian XX/XY are conserved in Madagascan chameleons of the genus Furcifer
Source: Sci Rep. 2024 Feb 28;14:4898. doi: 10.1038/s41598-024-55431-9 (PMC10901801; doi:10.1038/s41598-024-55431-9)
Supplement: Supplementary file 1 — Supplementary Legends. [file 41598_2024_55431_MOESM1_ESM.docx]

**Table S1:** Geneious Prime mapping parameters.

**Table S2:** Analysis of microdissected sex chromosomes.

**Table S3:** Comparative coverage analysis in *F. lateralis* and *F. pardalis*.

**Table S4:** Primers and qPCR values.
